# Heart Rate Measures From Wrist-Worn Activity Trackers in a Laboratory and Free-Living Setting: Validation Study

**DOI:** 10.2196/14120

**Published:** 2019-10-02

**Authors:** Andre Matthias Müller, Nan Xin Wang, Jiali Yao, Chuen Seng Tan, Ivan Cherh Chiet Low, Nicole Lim, Jeremy Tan, Agnes Tan, Falk Müller-Riemenschneider

**Affiliations:** 1 Health Systems & Behavioral Sciences Saw Swee Hock School of Public Health National University of Singapore and National University Health System Singapore Singapore; 2 Centre for Sport & Exercise Sciences University of Malaya Kuala Lumpur Malaysia; 3 Saw Swee Hock School of Public Health National University of Singapore and National University Health System Singapore Singapore; 4 Yong Loo Lin School of Medicine National University of Singapore and National University Health System Singapore Singapore; 5 Department of Physiology Yong Loo Lin School of Medicine National University of Singapore Singapore Singapore; 6 Health Promotion Board Singapore Singapore Singapore; 7 Institute for Social Medicine, Epidemiology and Health Economics Charite University Medical Centre Berlin Berlin Germany

**Keywords:** eHealth, mHealth, wearable, exercise, measurement, fitness, public health, quantified self

## Abstract

**Background:**

Wrist-worn activity trackers are popular, and an increasing number of these devices are equipped with heart rate (HR) measurement capabilities. However, the validity of HR data obtained from such trackers has not been thoroughly assessed outside the laboratory setting.

**Objective:**

This study aimed to investigate the validity of HR measures of a high-cost consumer-based tracker (Polar A370) and a low-cost tracker (Tempo HR) in the laboratory and free-living settings.

**Methods:**

Participants underwent a laboratory-based cycling protocol while wearing the two trackers and the chest-strapped Polar H10, which acted as criterion. Participants also wore the devices throughout the waking hours of the following day during which they were required to conduct at least one 10-min bout of moderate-to-vigorous physical activity (MVPA) to ensure variability in the HR signal. We extracted 10-second values from all devices and time-matched HR data from the trackers with those from the Polar H10. We calculated intraclass correlation coefficients (ICCs), mean absolute errors, and mean absolute percentage errors (MAPEs) between the criterion and the trackers. We constructed decile plots that compared HR data from Tempo HR and Polar A370 with criterion measures across intensity deciles. We investigated how many HR data points within the MVPA zone (≥64% of maximum HR) were detected by the trackers.

**Results:**

Of the 57 people screened, 55 joined the study (mean age 30.5 [SD 9.8] years). Tempo HR showed moderate agreement and large errors (laboratory: ICC 0.51 and MAPE 13.00%; free-living: ICC 0.71 and MAPE 10.20%). Polar A370 showed moderate-to-strong agreement and small errors (laboratory: ICC 0.73 and MAPE 6.40%; free-living: ICC 0.83 and MAPE 7.10%). Decile plots indicated increasing differences between Tempo HR and the criterion as HRs increased. Such trend was less pronounced when considering the Polar A370 HR data. Tempo HR identified 62.13% (1872/3013) and 54.27% (5717/10,535) of all MVPA time points in the laboratory phase and free-living phase, respectively. Polar A370 detected 81.09% (2273/2803) and 83.55% (9323/11,158) of all MVPA time points in the laboratory phase and free-living phase, respectively.

**Conclusions:**

HR data from the examined wrist-worn trackers were reasonably accurate in both the settings, with the Polar A370 showing stronger agreement with the Polar H10 and smaller errors. Inaccuracies increased with increasing HRs; this was pronounced for Tempo HR.

## Introduction

The scientific evidence on the health and well-being benefits of physical activity (PA) is overwhelming, and, as such, increasing activity levels is a core public health target [1,2]. The greatest benefits are attained when engaging in regular moderate-to-vigorous PA (MVPA). For example, positive effects of MVPA have been shown in the domains of mortality risk [3,4] as well as physical and psychological health and well-being [1,5-7].

The PA research landscape could broadly be divided into 2 core facets—PA surveillance and PA promotion [1,2]. Key to progress in both is the accurate measurement of PA. In this regard, questionnaires that are prone to recall and social desirability bias [8] are increasingly being complemented by instruments that measure PA objectively. Wearable research-grade devices such as accelerometers are commonly used, which have improved the validity of PA estimates [9,10]. Unfortunately, conducting population-wide studies with accelerometers is difficult because of high costs and participant burden.

To this end, the soaring availability and use of commercial wrist-worn activity tracking devices are increasingly being harnessed by PA researchers who are keen to use them for large-scale surveillance and intervention studies [11-13]. Sensor technologies inbuilt in these trackers allow for the convenient collection of various types of data [14]. As such, observational research and PA monitoring in intervention studies might soon primarily rely on data collected through devices that were not developed for research purposes. However, to make adequate use of wrist-worn tracker data, the validity of such data needs to be established [15]. There are numerous validation studies that have been conducted in recent years, and most of the studies focused on the accuracy of accelerometer-based metrics (eg, step counts) that are available from generation 1 activity trackers [16-18].

In addition to measurements of accelerometer-based metrics, many newer wrist-worn trackers are equipped with capabilities to collect data on physiological measures such as heart rate (HR) [14]. Estimating HR is enabled through photoplethysmography (PPG), a technology that consists of light-emitting diodes and photodetectors. With this, volume changes in the pulsatile component in the microvascular bed of arterial blood can be captured through reflection of the emitted light through the tissue [19]. Algorithms are then applied to estimate HR from PPG information. The 2 key advantages to measuring HR instead of, or in addition to, other metrics are the capture of nonweight-bearing activities (eg, cycling) and the ability to ascertain PA intensity, which is important for MVPA monitoring.

Validating HR data from wrist-worn trackers in healthy individuals is a recent endeavor [14]. Despite the increasing research activity, there is currently little uniformity in the technologies used and the conditions in which studies have been conducted. For example, although most researchers assessed the accuracy of tracker-based HR data during cycling or treadmill exercises [20-36], protocols varied widely (eg, different speeds and varying durations). Some of these studies also examined tracker accuracy during chores [31], outdoor activities [33], and resistance exercises [32,35,36], and 1 research team was solely interested in HR data accuracy during sedentary time [37]. As such, drawing firm and generalizable conclusions about tracker accuracy in terms of HR data is difficult, and interested readers are advised to consult studies that assessed the accuracy of specific trackers during specific activities (eg, accuracy of the various Fitbit devices (Fitbit, Inc) during cycling).

What the above-mentioned studies have in common is that they were conducted in a controlled laboratory setting. However, there are differences between a controlled and less controlled environment, and collecting data in both environments is warranted to disentangle such differences and increase ecological relevance of findings. To our knowledge, there are only 2 free-living studies. One research team merely collected HR data during common daily activities over a few hours [38], whereas the other included only 1 participant [39]. This is unsatisfying because wrist-worn trackers are meant to accurately capture HRs in different PA intensity zones (eg, light PA and MVPA) throughout the day and in different people. In addition, sample sizes were mostly small, with only few studies having more than 50 participants [22,28,40]. Finally, HR validation studies have so far only included devices that are rather expensive (mainly devices from Fitbit; 40 of 61 validation studies) [14]. With this, many people who might benefit from trackers will not be able to afford them. Less expensive trackers are readily available, but they are rarely tested. If these more affordable devices are reasonably accurate, large-scale studies and population-based health promotion campaigns using such activity trackers could become commonplace.

This study aimed to examine the validity of HR data from 2 wrist-worn HR trackers, the Tempo HR, a low-cost device used for a national PA promotion campaign in Singapore, and the Polar A370, a consumer-based fitness and activity tracking device, in laboratory and free-living settings. Both these trackers have not been assessed previously.

## Methods

### Design

We conducted a 2-phased validation study with all participants: laboratory phase and free-living phase. The study procedures were approved by the institutional review board of the National University of Singapore (NUS IRB: S-18-026), and written-informed consent was obtained from all participants before study enrolment. Data collection took place between March and May 2018.

### Participants

We applied multiple recruitment strategies to ensure a sample with varied characteristics. Students and staff were recruited through a post on the university’s Web-based learning system blackboard and word-of-mouth. Participants from the general public were recruited through emails sent to participants of the National Steps Challenge (NSC), a national PA promotion campaign rolled out by the Health Promotion Board (HPB), Singapore, yearly for 6 months (October to April).

Interested people were assessed for eligibility during an initial screening call and during the laboratory visit. The following inclusion criteria were applied: reasonably physically active English-literate men and women aged between 21 and 50 years with a body mass index (BMI) of at least 18.5 kg/m^2^; absence of physical disabilities or illness that would restrict moderate PA as assessed with the Physical Activity Readiness Questionnaire [41]; ownership of a mobile phone that supports HPB’s Healthy365 app, which was needed for data retrieval from the Tempo HR tracker, and that is compatible with HPB’s activity trackers; and willingness to use the personal mobile phone. Participants were instructed to abstain from caffeine for 12 hours and from food for 2 hours before the first study center visit.

### Procedures: Laboratory Phase

During the first visit, we collected sociodemographic information and measured height and weight with a SECA stadiometer (SECA GmbH). Following this, participants were fitted with 3 HR monitoring devices. We used the chest-strapped Polar H10 HR monitor (Polar Electro Oy) as our criterion device. Concurrent validity of similar Polar devices against echocardiogram (ECG) is well established [42]. The device was placed below the chest muscles. It transmitted real-time HR data to a wristwatch via Bluetooth. Our 2 wrist-worn HR trackers were the trackers used for the NSC (Tempo HR, J-style, TEMPO) and the Polar A370 (Polar Electro Oy). The Tempo HR is a low-cost activity tracker that measures steps, distance, calories burnt, and HR. Data from this tracker were transferred to the participants’ Healthy365 app, downloaded by HPB staff at the backend before it was shared with the researchers. Those recruited through HPB were already in possession of the Tempo HR tracker. The Polar A370 is a commercial activity tracker that allows monitoring of steps, distances, pace, global positioning system location, calories burnt, and HR. Data were transferred to the associated Polar Flow app and downloaded to the computer. Devices were worn snugly on opposite wrists (Tempo HR: left and Polar A370: right, during both the phases). Resting HR following at least 5 min of continuous sitting was measured before the cycling protocol.

Participants were requested to go through an incremental cycling protocol of 20 min on a stationary exercise bicycle (Monark 894E). The protocol consisted of four 5-min stages, and participants were required to cycle at an intensity corresponding to their designated HR zones for each stage (45%, 55%, 65%, and 75% of maximum HR [HR_max_]; ±10 beats per minute [bpm]) [22]. HR_max_ was calculated according to the common formula 220−age in years [43]. During the cycling program, researchers monitored adherence to the HR zones, provided verbal encouragement if necessary, and recorded perceived exertion at midpoint of each stage using the well-established 15-point visual Borg scale [44]. Following the cycling program, participants’ recovery HR was monitored for 5 min.

### Procedures: Free-Living Phase

After completing the cycling protocol, participants were introduced to the procedures of the free-living phase. In addition to the devices used in the laboratory phase, we provided participants with an ActiGraph wGT3X+BT accelerometer (ActiGraph) to collect HR data from the Polar H10 chest strap via Bluetooth. The small tamper-proof device was attached with a belt to the right side of the hip. We also provided an instruction sheet detailing adequate wear.

Participants were instructed to wear the devices during waking hours of the following day (after getting up in the morning until bedtime at night) and only remove them during water-based activities. In addition, we requested that participants engage in at least one 10-min bout of MVPA during the day to capture a wide range of HR signals. Finally, participants were provided with a device-wear log to record their wear and nonwear as well as their MVPA session(s). Participants returned to the laboratory a few days later to return the study devices and transfer HR data of the Tempo HR to the Healthy365 app.

### Data Acquisition and Synchronization

The sampling frequencies of the Tempo HR, Polar A370, and Polar H10 chest strap were 0.1 Hz, 1 Hz, and 1 Hz, respectively. As such, HR data were collected every second by the Polar devices and every 10 seconds by the Tempo HR (a sample of the raw data is provided in Multimedia Appendix 1). All devices provided time-stamped HR data based on the Network Time Protocol (GMT plus 8 hours). This allowed for time matching of data. For our analyses, we extracted the 10-second values from all 3 devices and time matched the nonzero HR data from Tempo HR and Polar A370 with those from Polar H10. The following data inclusion criteria were applied for the 2 phases separately: availability of at least 10 min of time-matched data for the laboratory phase and availability of at least 180 min of time-matched data for the free-living phase.

### Statistical Analysis

We summarized participants’ characteristics descriptively using mean and SD for continuous variables and number and percentage for categorical variables.

We calculated the intraclass correlation coefficients (ICCs) using mixed effects models to assess the absolute agreement between the criterion (Polar H10) and the other trackers (Tempo HR and Polar A370) in the laboratory phase and free-living phase. The strength of the ICC was interpreted as weak (<0.50), moderate (≥0.50 to 0.74), strong (≥0.75 to 0.89), and very strong (≥0.90) [45]. To facilitate visual inspection, we created scatterplots of HRs between devices with all participants and similarly for each participant (not shown).

We then calculated mean absolute errors (MAEs) and mean absolute percentage errors (MAPE; absolute error/criterion×100) between the criterion (Polar H10) and, both, the Tempo HR and the Polar A370 trackers, to gauge overall measurement error. As highlighted in a recent study, there is no clear cutoff for what level of error would indicate adequate validity between measures [32]. After considering the available options and similar to the authors of a previous study, we adopted a cutoff of 10% to judge validity [46], a cutoff that also coincides with the one suggested by the Association for the Advancement of Medical Instrumentation in their document on the validity of HR measurement devices [47]. Bland-Altman (BA) plots with limits of agreement (LoA) set at 95% were used to visualize agreement and proportional bias.

Moreover, we ranked the 10-second HR time points derived from the Polar H10 and divided them into deciles. As such, decile 1 contained the lowest 10% of all HR and decile 10 contained the highest 10% of all HR. We then time matched these HR deciles with HR data from the Tempo HR and Polar A370. We constructed the box plots to compare the HR data from the Tempo HR and the Polar A370 with the Polar H10 measures across the deciles.

Finally, we constructed 2×2 tables to estimate the sensitivity and specificity of the 2 trackers for identifying the different HR zones based on the Polar H10 (<64% HR_max_ and ≥64% HR_max_). The cutoff of 64% HR_max_ was chosen because it is the updated cutoff [48] of the earlier 50% HR_max_ cutoff [49]. The more recent cutoff has since been endorsed by the American College of Sports Medicine [50]. All statistical analyses were conducted using R (version 3.4.2).

## Results

### Study Participants

**Figure 1:**
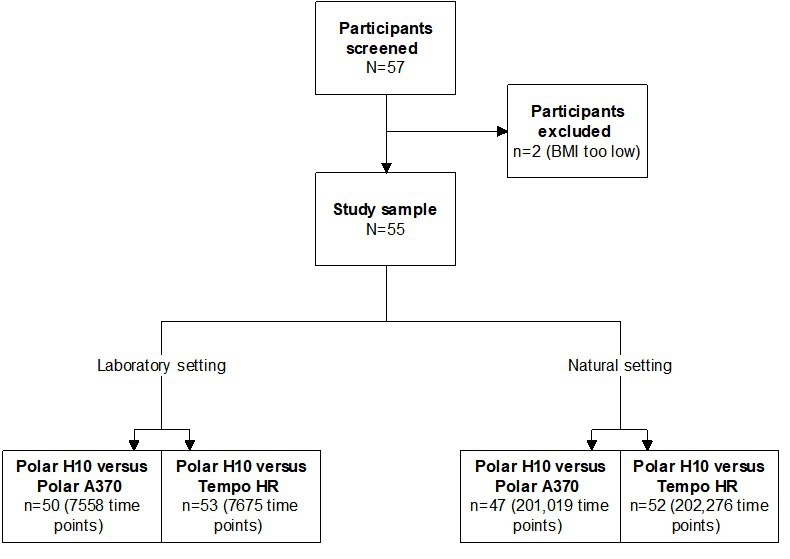
Data analysis flow showing participants in analysis (n) and number of matched heart rate time points. BMI: body mass index; HR: heart rate.

Of the 57 people screened, 55 were eligible and joined the study (mean age 30.5 [SD 9.8] years), with 26 being female (47%), 36 with normal weight (65%; BMI <23 kg/m^2^), and 39 with Chinese ethnicity (71%). Due to the unavailability of some HR data, few participants were excluded from some analyses. Figure 1 depicts the analysis flow, which also indicates data availability. During the free-living phase, and after excluding data points with zero measures, mean wear time of the Tempo HR, Polar A370, and Polar H10 was 12.2 (SD 2.6) hours, 12.8 (SD 2.7) hours, and 11.7 (SD 3.1) hours, respectively.

### Overall Agreement

#### Laboratory Phase

In the laboratory phase, the HR data from the Tempo HR showed a moderate ICC (0.51; 95% CI 0.38 to 0.60) with the data from Polar H10. With a MAE of 15.1 bpm (95% CI 14.6 to 15.5 bpm) and an MAPE of 13.0%, the measurement error was somewhat large. Polar A370 data also had a moderate but stronger ICC with the Polar H10 (0.73; 95% CI 0.66 to 0.78). Measurement errors were small with a MAE of 7.3 bpm (95% CI 7.0 to 7.7 bpm) and an MAPE of 6.4%. On average, both the devices underestimated HR: Tempo HR by 9.7 bpm (95% CI −10.2 to −9.2 bpm) and Polar A370 by 5.7 bpm (95% CI −6.1 to −5.3 bpm).

Figure 2 shows the BA plot, and Figure 3 shows the HR decile plot between the Tempo HR and the Polar H10. These plots showcase 3 trends: HR tends to be underestimated by the Tempo HR across the range of HR values; the increase in HR is accompanied by an increasing difference between the Tempo HR and Polar H10 HR data; and the variability of the HR data from the Tempo HR increases with increasing HR and the variability is especially pronounced at the higher HR deciles (Figure 3).

**Figure:**
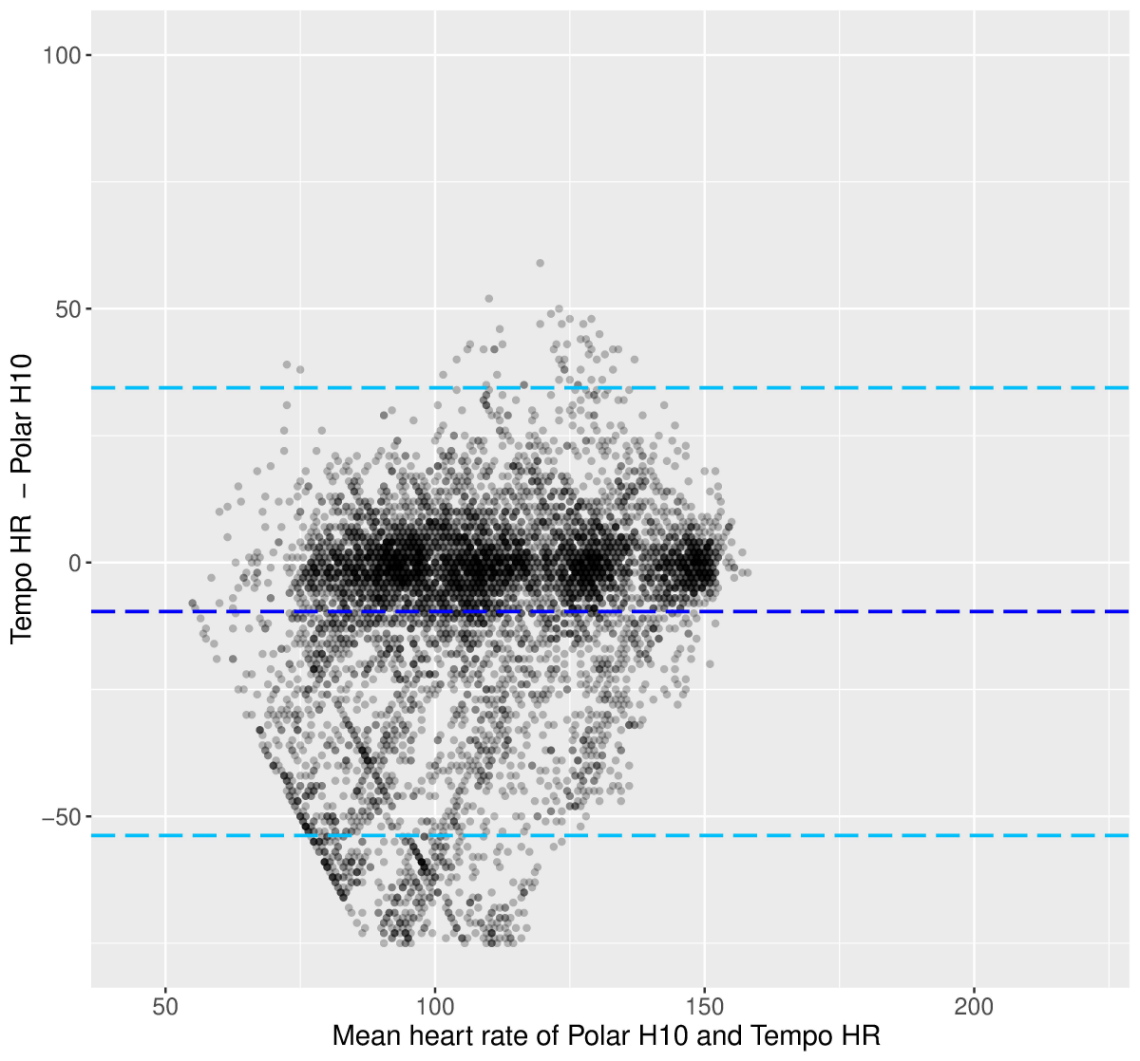
Laboratory phase: Bland-Altman plot between the heart rate data from the Polar H10 and the Tempo HR. Light blue dotted lines show the limits of agreement, and the dark blue dotted line shows the mean of the difference. HR: heart rate.

**Figure:**
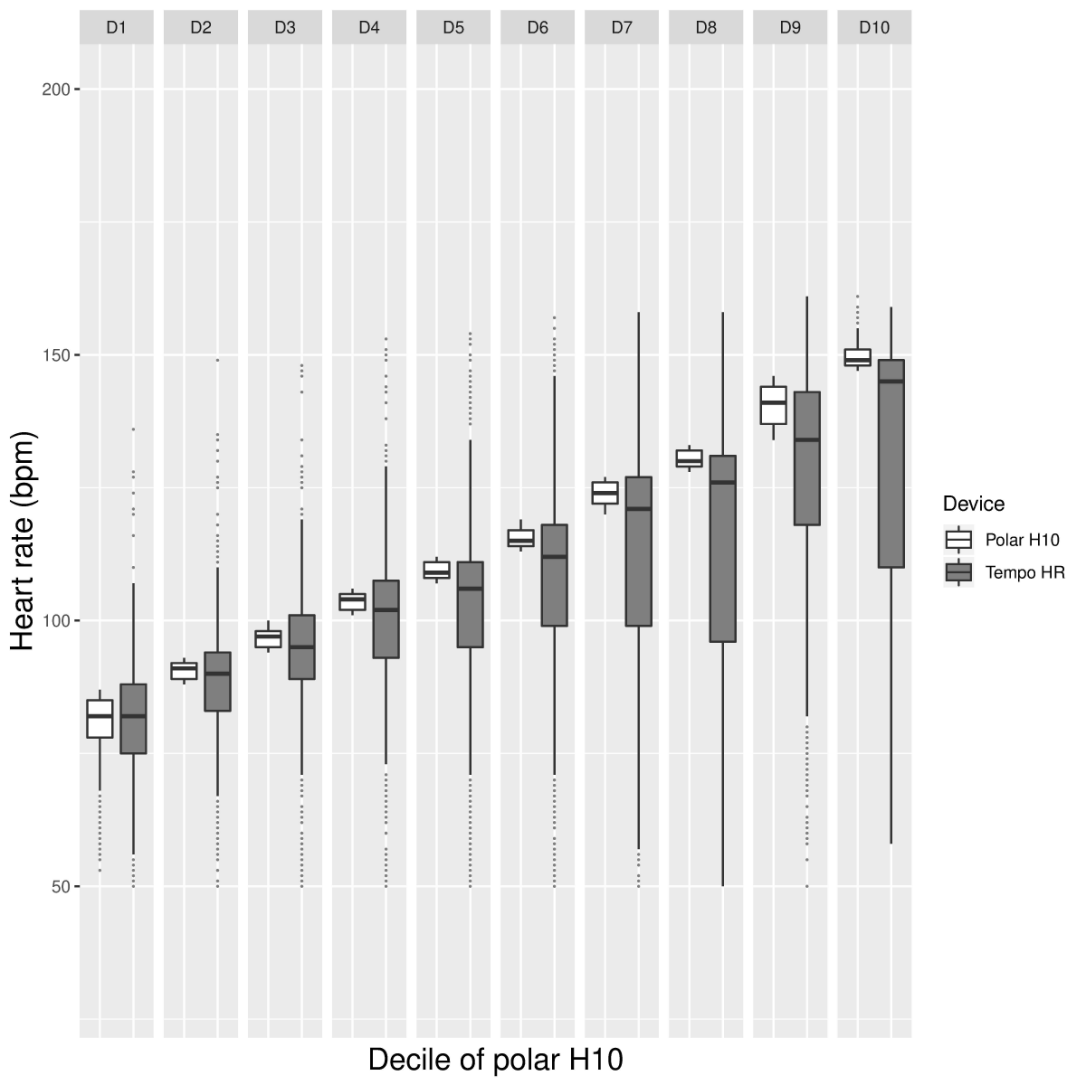
Laboratory phase: box plot providing by-decile comparisons of mean Polar H10 (white) and Tempo HR HR data (gray). HR: heart rate; bpm: beats per minute.

As can be seen in Figures 4 and 5, the trends described above are less pronounced when considering HR data from the Polar A370 tracker. First, it can be seen that the underestimation of HR is occurring across HR values (Figure 4). Second, the decile plot does not indicate a marked change in the difference between the data from the Polar A370 and the Polar H10 across HRs. Third, the variability of Polar A370 does not increase markedly with increasing HR (Figure 5).

**Figure:**
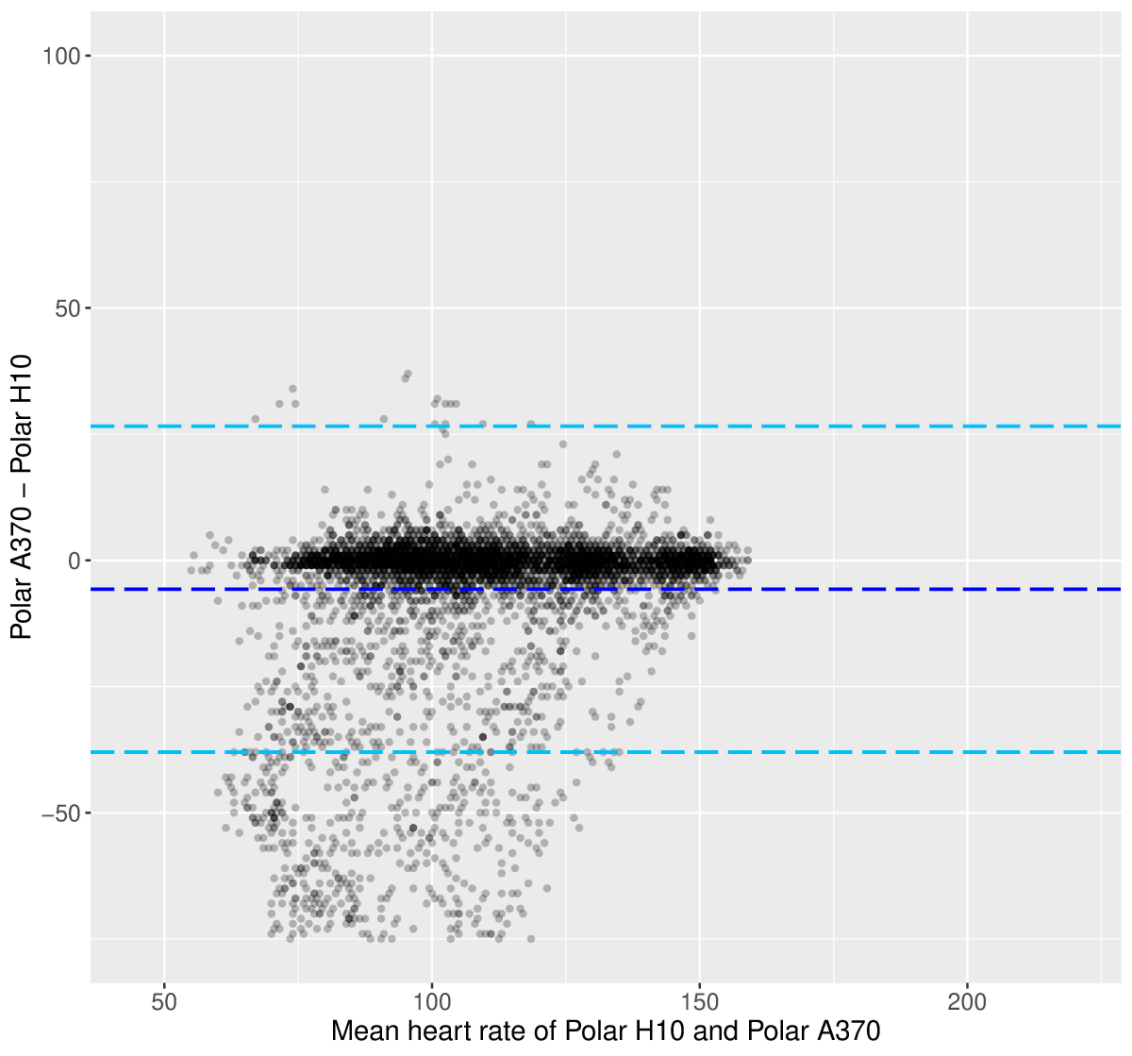
Laboratory phase: Bland-Altman plot between the heart rate data from the Polar H10 and the Polar A370. Light blue dotted lines show the limits of agreement, and the dark blue dotted line shows the mean of the difference. HR: heart rate.

**Figure:**
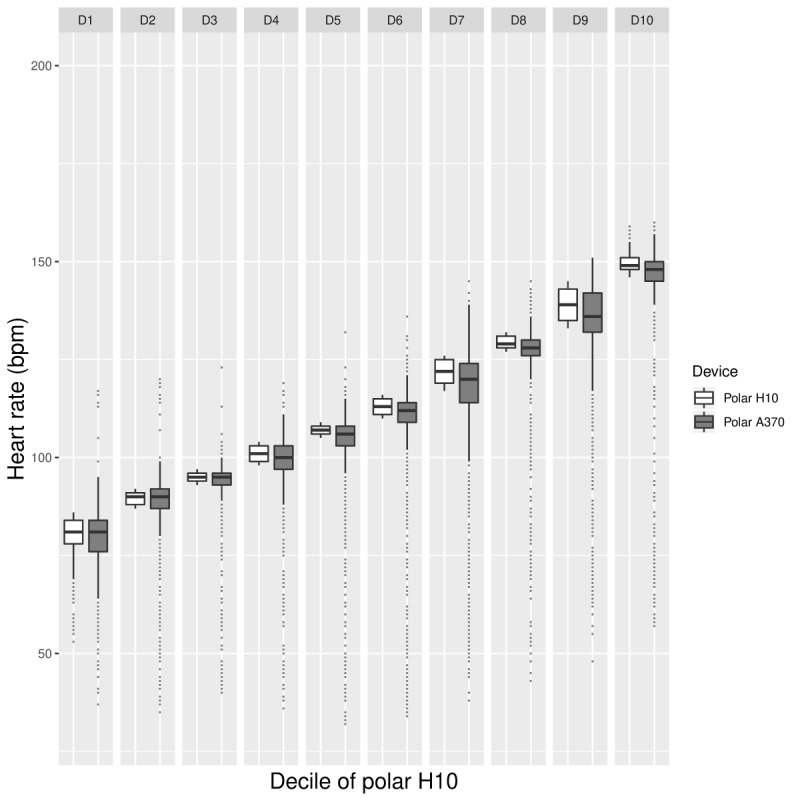
Laboratory phase: box plot providing by-decile comparisons of mean Polar H10 (white) and Polar A370 HR data (gray). HR: heart rate; bpm: beats per minute.

#### Free-Living Phase

The ICC between the Polar H10 and the Tempo HR data was moderate in the free-living phase (0.71; 95% CI 0.70 to 0.71). Errors were smaller compared with the laboratory phase with a MAE of 8.7 bpm (95% CI 8.7 to 8.8 bpm) and an MAPE of 10.2%. For the Polar A370, the ICC between the Polar H10 and the Polar A370 tracker data was strong (0.83; 95% CI 0.79 to 0.87). Errors were similar compared with the ones in the laboratory phase with a MAE of 5.9 bpm (95% CI 5.8 to 5.9 bpm) and an MAPE of 7.1%. In contrast to the results from the laboratory phase, both the devices overestimated HR slightly (Tempo HR 0.4 bpm; 95% CI 0.3 to 0.5 bpm and Polar A370 3.4 bpm; 95% CI 3.3 to 3.4 bpm).

The BA plot in Figure 6 depicts the potential occurrences of overestimation and underestimation of HR measures from the Tempo HR across HR values. Although no clear trend can be established, it appears that HR overestimation is more common. As shown in Figure 7, overestimation tends to occur at lower HRs, whereas underestimation happens more frequently at higher HRs. In addition, the decile plot shows that the HR difference between the Polar H10 and the Tempo HR is minimal until decile 8 where it begins to increase markedly. At decile 10, the difference is substantial. In addition, Tempo HR data vary to a similar degree until decile 10, where the variability is high.

Figure 8 shows the BA plot, and Figure 9 shows the HR decile plot between the Polar A370 and the Polar H10. Both plots indicate that the Polar A370 appears to overestimate HR at lower HRs (below decile 9). However, the decile plot shows that the overall difference between the criterion and the Polar A370 is not substantial throughout. As for the Tempo HR, data variability is high only in decile 10.

**Figure:**
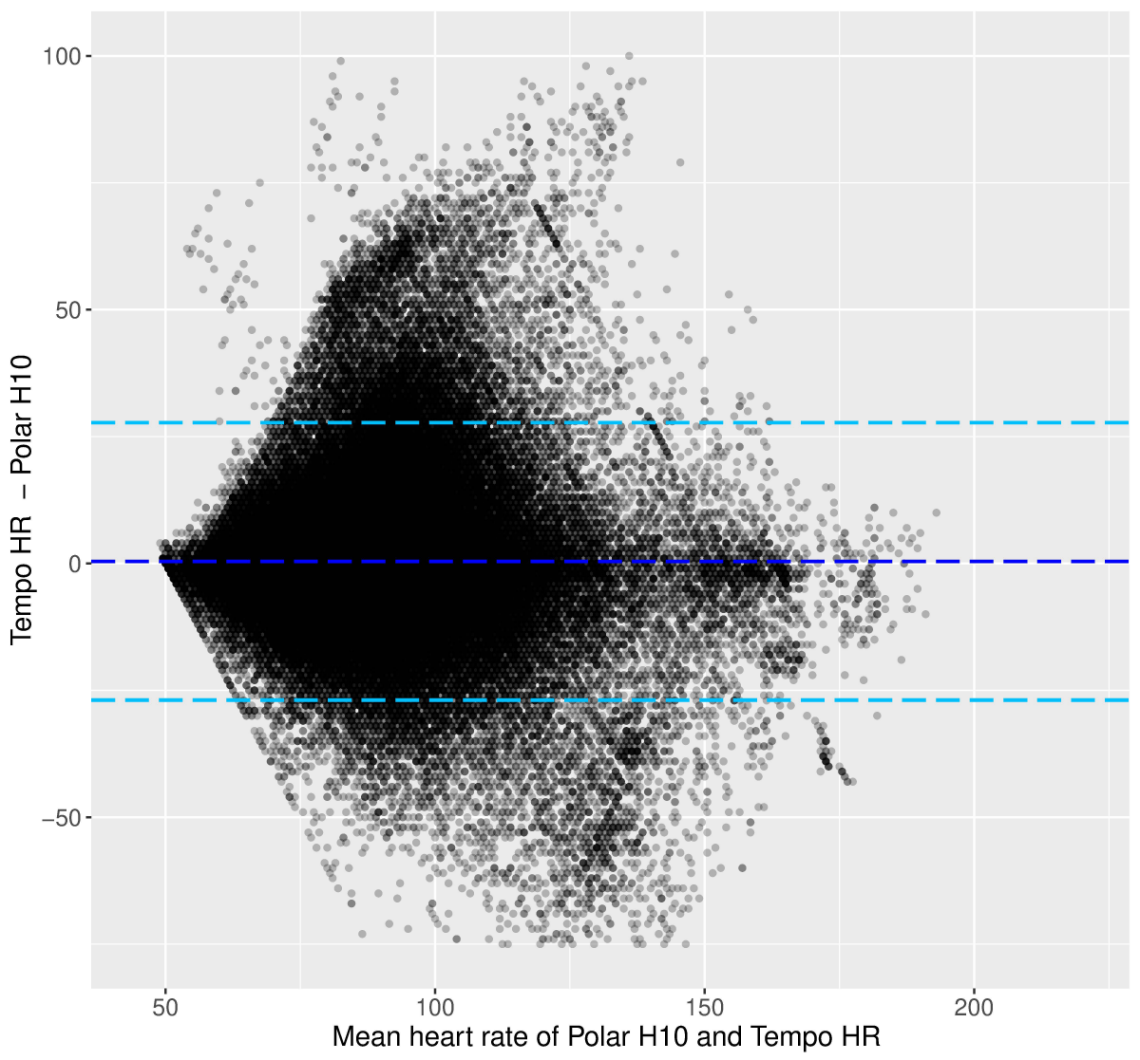
Free-living phase: Bland-Altman plot between the heart rate data from the Polar H10 and the Tempo HR. Light blue dotted lines show the limits of agreement, and the dark blue dotted line shows the mean of the difference. HR: heart rate.

**Figure:**
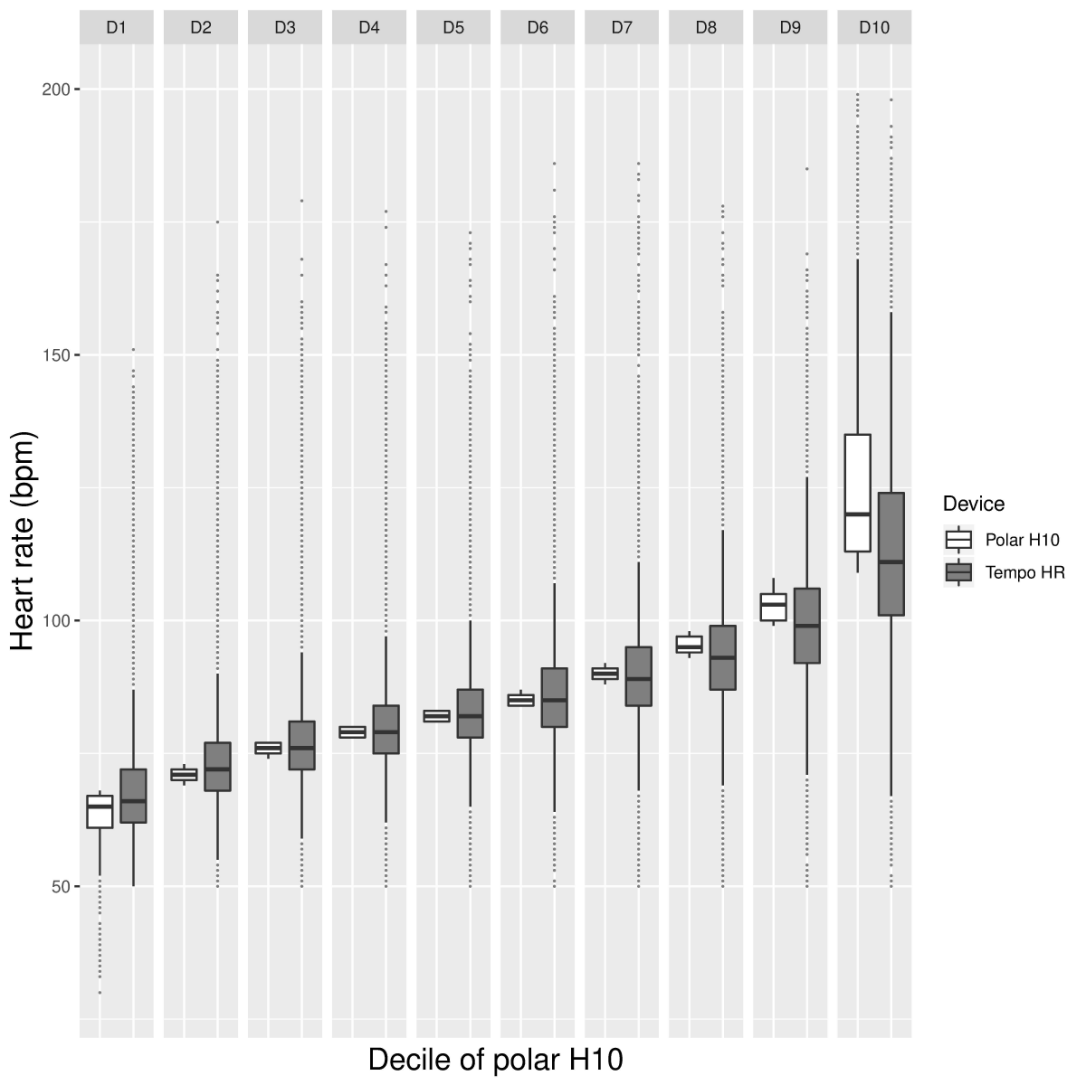
Free-living phase: box plot providing by-decile comparisons of mean Polar H10 (white) and Tempo HR HR data (gray). HR: heart rate; bpm: beats per minute.

**Figure:**
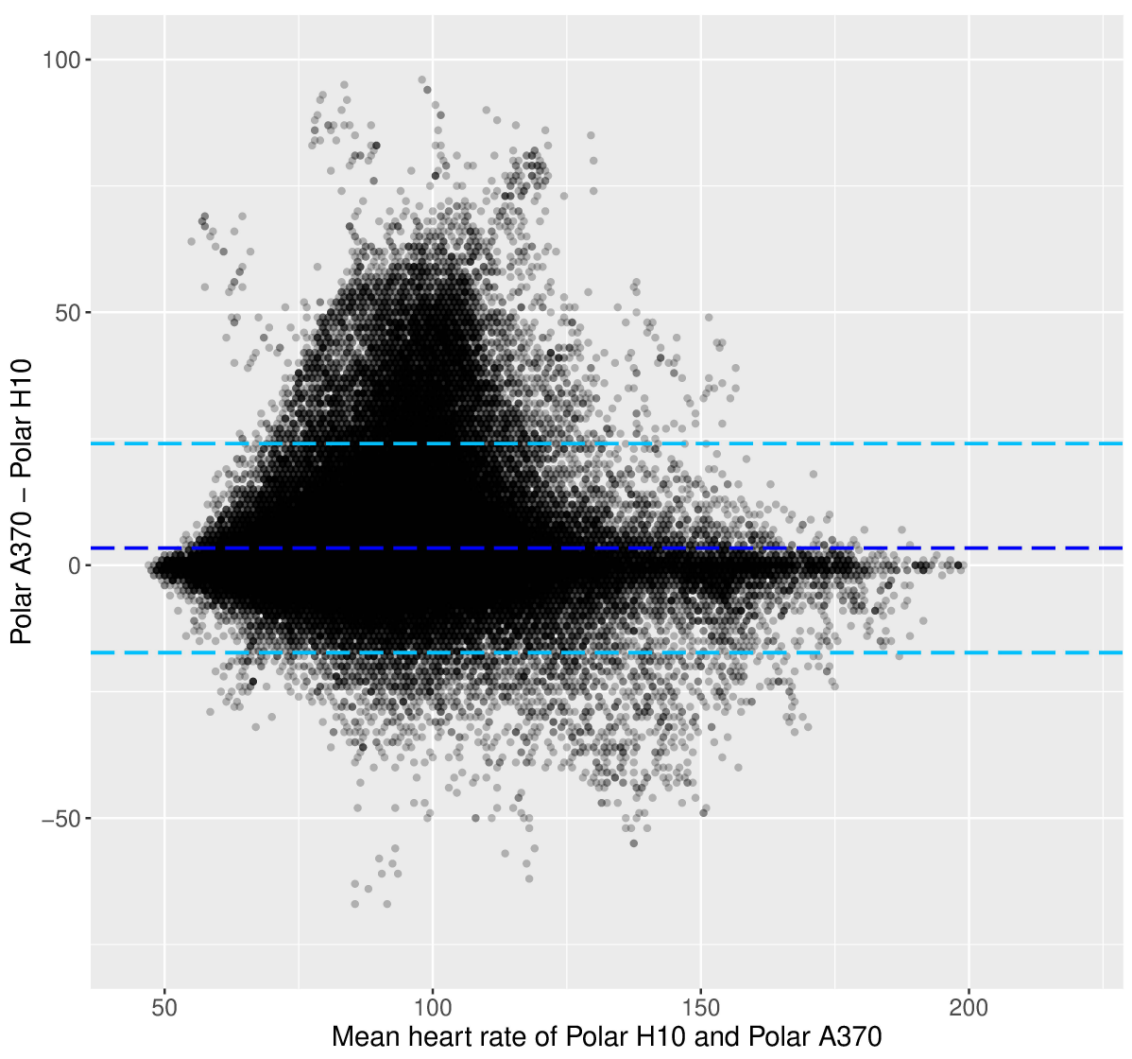
Free-living phase: Bland-Altman plot between the heart rate data from the Polar H10 and the Polar A370. Light blue dotted lines show the limits of agreement, and the dark blue dotted line shows the mean of the difference. HR: heart rate.

**Figure:**
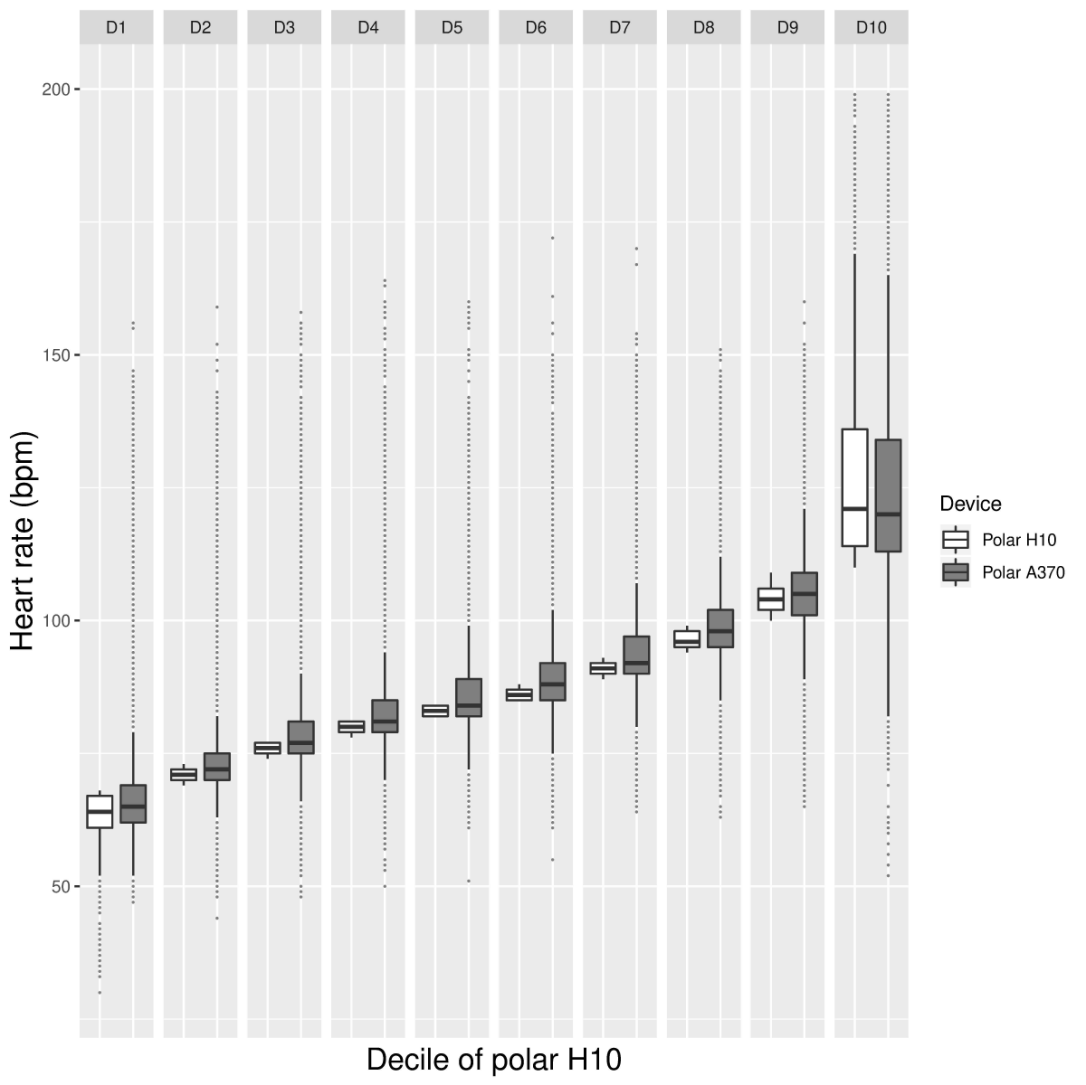
Free-living phase: box plot providing by-decile comparisons of mean Polar H10 (white) and Polar A370 HR data (gray). HR; heart rate; bpm: beats per minute.

### Sensitivity and Specificity

When analyzing how many MVPA time points were identified by the Tempo HR and the Polar A370, we set the MVPA cutoff at 64% HR_max_. In the laboratory phase, of the total aggregate time points in the MVPA HR zone that were detected by the Polar H10, 62.13% (1872/3013) were also identified by the Tempo HR, whereas the Polar A370 identified 81.09% (2273/2803). The remaining time was spent below the MVPA HR zone, of which 91.52% (4267/4662) and 97.52% (4637/4755) were also registered by the Tempo HR and the Polar A370, respectively. Overall, the Tempo HR identified 79.99% (6139/7675) and the Polar A370 91.42% (6910/7558) of data points accurately.

In the free-living phase, we found that the Tempo HR identified 54.27% (5717/10,535) and the Polar A370 identified 83.55% (9323/11,158) of the MVPA time points that the Polar H10 registered. The Tempo HR picked up 97.22% (186,402/191,741) and the Polar A370 picked up 96.72% (183,625/189,861) of time points below the MVPA HR zone. Overall accuracy was above 90% for both the trackers (Tempo HR: 94.98%, 192,119/202,276; Polar A370: 95.98%, 192,948/201,019). An overview of the results is provided in Table 1.

**Table 1:** Number of 10-second matched time points spent in heart rate zones as detected by the Polar A370 and the Tempo HR in the laboratory phase and free-living phase.

According to Polar H10			≥64% HR_max_^a^, n (%)	<64% HR_max_, n (%)
**Laboratory phase**				
	**According to Polar A370**			
		≥64% HR_max_	2273 (81.09)	118 (2.48)
		<64% HR_max_	530 (18.91)	4637 (97.52)
	Total		2803 (37.09)	4755 (62.91)
	**According to Tempo HR**			
		≥64% HR_max_	1872 (62.13)	395 (8.47)
		<64% HR_max_	1141 (37.87)	4267 (91.53)
	Total		3013 (39.26)	4662 (60.74)
**Free-living phase**				
	**According to Polar A370**			
		≥64% HR_max_	9323 (83.55)	6236 (3.28)
		<64% HR_max_	1835 (16.45)	183,625 (96.72)
	Total		11,158 (5.55)	189,861 (94.45)
	**According to Tempo HR**			
		≥64% HR_max_	5717 (54.27)	5339 (2.78)
		<64% HR_max_	4818 (45.73)	186,402 (97.22)
	Total		10,535 (5.21)	191,741 (94.79)

^a^HR_max_: maximum heart rate.

## Discussion

### Principal Findings

From the present 2-phased tracker validation study involving 55 participants with varying characteristics, a few key findings can be highlighted. First, HR data from the low-cost Tempo HR tracker showed moderate agreement with the data from the chest-strapped Polar H10 in both the laboratory phase and free-living phase. Although the measurement errors of the Tempo HR were above the 10% validity cutoff [46,47] in both phases, indicating its limited validity when measuring HR, the measurement error was markedly lower and close to the cutoff in the free-living phase (10.2% error). Second, HR data from the consumer-based Polar A370 showed strong agreement with data from the Polar H10 and low measurement errors (below the 10% validity cutoff) in both phases. Third, the differences between the Tempo HR and the Polar H10 are highest at higher HRs in both phases. This suggests that the measurement errors highlighted above are mainly the result of errors at high HRs. Further evidence for this conclusion can be derived from the sensitivity and specificity analysis where the Tempo HR identified only more than 50% of HRs above the MVPA threshold in both phases, whereas the Polar A370 identified more than 80% of HRs above the MVPA threshold in both phases. Fourth, agreement was generally higher and errors were smaller in the free-living phase compared with the laboratory phase. Finally, both trackers underestimated HR in the laboratory phase, whereas they overestimated it slightly in the free-living phase.

To establish the stability of the study results, we conducted sensitivity analyses. For this, we removed outliers and compared Polar H10 with the 2 other trackers using the remaining matched data points available. Outliers were defined as follows: a Pearson correlation coefficient of less than 0.3 between the Polar H10 and the test trackers in the laboratory setting. In secondary analyses, we only used data that were available from all 3 devices. Conducting these analyses did not change the results markedly (data not shown). As such, the reported results are not influenced by extreme cases or outliers.

### Heart Rate Accuracy of the Polar A370 and Tempo HR in Context

When contextualizing our laboratory findings with those reported in the literature, the Polar A370 and the Tempo HR appear to have comparable or better accuracy with the market leader Fitbit, which has been studied extensively [20-25,28-32,34,36,37]. For example, authors who also asked participants to go through a cycling ergometer program reported agreement coefficients for Fitbit devices of between 0.21 and 0.50 [30,32,34]. The ICCs for the Polar A370 and Tempo HR in our study were 0.73 and 0.51, respectively. Similarly, other studies reported Fitbit MAPEs of 15.9% [34] and 21.06% [32], whereas the MAPE of the Polar A370 in our study was 6.4%; the one for the Tempo HR was 13.0%.

Comparing our results from the free-living phase with the results reported in other studies is problematic as, to our knowledge, there are only 2 studies that had a free-living element [38,39]. Gorny et al assessed data collected by the Fitbit Charge HR against data from the Polar H6 chest strap and reported an ICC of 0.83 and a mean difference between devices of −5.96 bpm. The ICC is in line with what we found for the Polar A370 in our study (ICC: 0.83). However, we observed overestimation in the free-living setting (Polar A370: 3.4 bpm; Tempo HR: 0.4 bpm). The authors also conducted sensitivity and specificity analysis and reported that the Fitbit Charge HR detected 52.9% of episodes spent in MVPA HR zones. Although this appears to be similar to what we found for the sensitivity of the Tempo HR (54.27%), the MVPA cutoffs in both studies were different. In our study, the more recent cutoff of 64% HR_max_ was used, whereas Gorny et al used the older 50% HR_max_ cutoff. One study by Nelson and Allen also provides some information on the accuracy of a Fitbit device in a free-living setting (Fitbit Charge 2). Over a 24-hour period, agreement measured by the concordance correlation coefficient was 0.91; this is close to what we found for the Polar A370 (although we used the ICC that provides similar estimates). MAE (4.9 bpm) and MAPEs (6.0%) for the Fitbit Charge 2 were also similar to that of the Polar A370 in our study (5.9 bpm, 7.1%). From these results, it appears that the Polar A370 is similarly accurate as the Fitbit Charge in free-living settings, whereas the Tempo HR appears to be less accurate.

The finding that the accuracy of wrist-worn trackers decreases as intensity increases has been observed in previous laboratory studies. For example, Boudreaux et al found that an increase in cycling intensity was associated with increasing HR underestimation in assessed activity trackers [32]. Dondzila et al made a similar discovery during treadmill exercises [21]. Spierer et al suggested that the increased measurement error with increasing movement intensity is because of increased motion, which leads to more disturbances of the blood flow-sensor interface [35].

It is difficult to draw firm conclusions about such trends in the free-living phase, as there are no comparable studies available. We observed smaller differences across activity intensities, which might be related to the fact that the proportion of higher HR values was rather small compared with the laboratory study. This might also partially explain the generally higher accuracy in the free-living phase versus the laboratory phase. Another reason for the difference in accuracy between the free-living phase and laboratory phase might be related to the temperature difference between the laboratory and the free-living settings [51]. The laboratory study was conducted in an air-conditioned environment in which the temperature varied between 18°C and 20°C. This is significantly colder than the outside temperature in Singapore (between 30°C and 32°C); hence, the free-living study was executed under warmer conditions. The fact that higher temperatures facilitate blood flow is well established. The aforementioned factors could also partially explain why the test devices underestimated HR in the laboratory phase and not in the free-living phase.

### Differences in Accuracy Between Devices

From the results of our study and the overall HR tracker validation literature, it is obvious that there are marked differences between devices in terms of accuracy that ought to be explained. A review by Tamura et al provides some insights into the factors that impact HR measurement through PPG in different devices [19]. First, PPG-measured HR differences between devices might be related to the algorithms used to estimate HR. Different devices use different algorithms for translating the detected blood flow into HR. A recent study highlighted that sensor technologies to detect physiological parameters are mostly identical between devices. However, the algorithms applied to translate the collected data into a readable HR measure vary from vendor to vendor and can be changed without notice [14]. Similarly, algorithms used to correct for movement artifacts during upper body movements vary between devices [52]. As such, it is the algorithm and not the technology itself that seems to primarily impact device accuracy. A second reason for the observed differences in accuracy between devices could be related to the contact force between the sensor and the skin [53]. Insufficient contact pressure is related to less sensitivity in detecting blood flow. Although both trackers were fitted snuggly (this was tested), the Polar A370 had more bracelet holes, which meant that its sensor might have had slightly better contact with the skin.

### Strengths and Limitations

A number of strengths of this study can be highlighted. To the best of our knowledge, this is the first study that thoroughly investigated the validity of HR measures of modern wrist-worn activity trackers in 2 settings, the laboratory and daily life. Research on the real-world performance of activity trackers can advance the PA and exercise measurement field substantially as these trackers are meant to be used as people go about their normal lives. Second, our study sample size was relatively large and diverse, which is rare in validation studies. Third, we were able to collect temporally dense HR data from all devices (approximately 12 hours per device in the free-living phase), which allowed us to conduct in-depth analyses of tracker validity across varying HRs. The richness of data we collected stands in stark contrast to most previous studies that relied mainly on few data points, for example, at the end or midpoint of a stage in a cycling protocol [32]. Despite these strengths, a few limitations ought to be mentioned. First, we opted for a cycling protocol in our laboratory phase, which might not be optimal as participants bent their wrists when holding on to the handlebar. However, using other protocols, such as a treadmill program, as in other studies, is not optimal either, as upper body movements will lead to movement artifacts that are likely to impact HR measures of the wrist-worn trackers [19]. Second, compared with other researchers who investigated the validity of up to 8 activity trackers [32], we only used 2 trackers in our study. Although this might appear to be a significant shortcoming, we limited the number of trackers intentionally. We based our decision on a small internal pilot study during which we established that wearing many trackers in addition to a chest-strap HR monitor during a free-living study would be too burdensome for participants; as such, we were concerned about study compliance. Third, it was not possible to ensure participants wore the devices accurately during the free-living phase. Although we explained how the devices should be fitted, practiced the wear protocol with participants, and provided a step-by-step instruction sheet, it is possible that participants did not wear the devices appropriately. However, as the accuracy was generally higher in the free-living phase versus the laboratory phase, we believe that inappropriate wear did not introduce significant errors. Fourth, we predetermined the wearing side of the 2 trackers (Tempo HR: left and Polar A370: right). There is some debate about whether the side at which trackers are worn could influence the HR data collected. Some researchers predetermined wear side [21,30,31,36-38,40], whereas others used randomization procedures [20,22,24,29,32]. We believe our protocol did not introduce bias and base this assumption on a 2017 study in which researchers found that the wearing side (left or right) was not associated with differences in HR measurement error in 6 commercial trackers; in 1 device, there were some small differences [28]. Finally, we used a chest-strapped HR monitor as our criterion device for measuring HR. Although ECG might have been the more adequate criterion, Polar chest straps are generally accepted reference devices as they have adequate validity [42]. In addition, Polar chest straps were used in many previous studies, and they were the only feasible criterion in our free-living phase.

### Future Directions

A recent review highlighted the strong increase in the availability and use of wrist-worn activity trackers and identified 432 different activity trackers that belonged to 123 unique brands [14]. As such, researchers will increasingly make use of them [11,54]. A key promise of such wrist-worn trackers is that they can facilitate PA behavior change through self-monitoring and feedback, 2 well-established behavioral change techniques that are supposed to enable individuals to bridge the gap between current behavior and behavioral targets [55,56]. However, research evidence on the effectiveness of tracker-based self-monitoring and feedback in terms of PA is currently mixed [13,57]. The effect of tracker use on PA behavior might be moderated by actual wear time [58]. In addition to self-monitoring, activity trackers are proposed to be important for just-in-time adaptive interventions in which in-the-moment behavioral support is delivered based on real-life data. Step counts are most commonly used for this purpose, with HR data as a basis for just-in-time support and feedback being a viable option. Due to the ability of sensors to communicate sensor-collected information on PA intensity to mobile phone apps, real-time adaptations of feedback and support is possible. Such dynamic interventions are suggested to increase sustainable behavior change through effective engagement [54,59]. Research in this field is in its infancy, but important gains are being made. Finally, observational research is likely to be a great beneficiary of tracker devices as they can be used to collect long-term PA data in an unobtrusive and resource-effective way.

## Appendix

Multimedia Appendix 1 Example of raw data retrieved from the Polar H10, Polar A370, and Tempo HR.

## Abbreviations

BA: Bland-Altman
BMI: body mass index
bpm: beats per minute
ECG: echocardiogram
HPB: Health Promotion Board
HR: heart rate
HR_max_: maximum heart rate
ICC: intraclass correlation coefficient
LoA: limits of agreement
MAE: mean absolute error
MAPE: mean absolute percentage error
MVPA: moderate-to-vigorous physical activity
NSC: National Steps Challenge
PA: physical activity
PPG: photoplethysmography
